# Microstructural evolution during heating of CNT/Metal Matrix Composites processed by Severe Plastic Deformation

**DOI:** 10.1038/s41598-020-57946-3

**Published:** 2020-01-21

**Authors:** Katherine Aristizabal, Andreas Katzensteiner, Andrea Bachmaier, Frank Mücklich, Sebastian Suárez

**Affiliations:** 1 Chair of Functional Materials, Department of Materials Science, Saarland University, D-66123 Saarbrücken, Germany; 2 Erich Schmid Institute of Materials Science, Austrian Academy of Sciences, Jahnstrasse 12, A-8700 Leoben, Austria

**Keywords:** Engineering, Materials science

## Abstract

Carbon nanotube reinforced nickel matrix composites (Ni/CNT) with different CNT compositions were fabricated by solid state processing and subjected to severe plastic deformation (SPD) by means of high pressure torsion (HPT). A thorough study on the microstructural changes during heating and on the thermal stability was performed using differential scanning calorimetry (DSC), high temperature X-ray diffraction (HT-XRD) and electron backscattered diffraction (EBSD). Furthermore, the formation and dissolution of the metastable nickel carbide Ni_3_C phase was evidenced by DSC and HT-XRD in composites, where sufficient carbon atoms are available, as a consequence of irreversible damage on the CNT introduced by HPT. Finally, it was shown that the composites exhibited an improved thermal stability with respect to nickel samples processed under the same conditions, with a final grain size dependent on the CNT volume fraction according to a *V*_*CNT*_^−1/3^ relationship and that lied within the ultrafine grained range.

## Introduction

Ultrafine-grained (UFG) and nanocrystalline (NC) materials obtained by severe plastic deformation (SPD) have been subject of intensive study due to their interesting functional and enhanced mechanical properties^1,2^. However, the high amount of stored energy stemming from a large grain boundary area and a high density of lattice defects makes them thermally unstable at lower temperatures relative to the coarse grained counterparts^2,3^, which may be a limiting factor for potential applications. Many studies have been performed on the thermal stability of these materials in order to better understand the mechanisms involved and to develop ways to overcome this drawback^4–7^. It has been shown that the stored energy of defects increases with the imposed strain during SPD, which decreases the temperature for the onset of recovery of such defects^8^. Furthermore, it has been observed that the behavior during heating of NC and UFG materials differs significantly from that of coarse polycrystalline ones. For instance, NC and UFG show usually different behavior during grain growth at low temperatures (typically <300 °C) than at higher ones^9–15^. This is mainly because at low temperatures recovery and recrystallization processes occur, which exhibit different activation energies than grain growth processes taking place at higher temperatures. Additionally, NC and UFG may also exhibit abnormal grain growth^16^. A study from G. K. Rane *et al*. about the grain growth on NC Ni powder produced by ball milling^15^, showed that upon annealing, rapid grain growth occurs at the beginning with almost total annihilation of microstrain, where with longer annealing times, the course of grain growth depends on the initial microstructure, exhibiting linear growth in the whole temperature range studied (in the case of samples with the larger microstrain and narrower grain size distributions). Such effects were found to be incompatible with grain-boundary curvature driven growth, including the “generalized parabolic grain growth model”, i.e. $D{(t)}^{n}\,-\,D{(0)}^{n}\,=\,{k}_{1}t$, (where: D(t) is the grain size at a time t, D(0) is the initial grain size, k_1_ is the rate constant proportional to the grain boundary Mobility M and n is the grain growth exponent) and even a grain growth model including a growth retarding contribution from impurities along the grain boundaries (Zener-Drag), did not fit well the studied data^15^.

Some efforts to improve the thermal stability have been made, including the addition of alloying elements and second phases to metallic matrices^17–19^. Carbon Nanotubes (CNT) have proven to be promising reinforcing phase due to their outstanding physical properties. Therefore, the thermal stability of such composites processed by SPD is expected to increase with respect to their matrix counterparts. Nevertheless, it has been observed that processing CNT-reinforced metal matrix composites (MMC) has some technical limitations. For instance, applying extremely high strains may induce structural damage on the CNT, which might lead to their amorphization^20^. Albeit the technical issues stemming from the difficulty to disperse CNT agglomerates effectively due to strong Van der Waals interaction, this drawback can be solved, to some extent, by SPD of the CNT-MMC. In fact, it was observed that there is a minimum strain that should be applied in order to obtain a homogenous distribution of the CNT^21^. Even though impurities and second phases can improve the thermal stability of MMC processed by SPD, UFG and NC CNT-MMC obtained by high pressure torsion (HPT) of bulk samples, possess a high density of defects and large grain boundary area, and thus, a high stored energy^22^, which may render them highly unstable when subjected to heat treatments. It is therefore important to study the evolution of the microstructure during and upon heating of such composites in order to determine to what extent the CNT can stabilize the microstructure against grain growth.

In this work, a thorough study was performed on the microstructural changes during heating and post annealing of CNT/Ni matrix composites, increasing the CNT content and the equivalent strain, comparing the results to bulk Ni samples deformed by HPT with the same parameters. Different characterization techniques were used, such as Differential Scanning Calorimetry (DSC), High Temperature X-ray diffraction (HT-XRD), Transmission electron microscopy (TEM) and Electron Backscattered diffraction (EBSD).

## Materials and Methods

### Materials

By means of colloidal mixing, powder mixtures were obtained from the starting materials (i.e. MWCNT - CCVD grown, Graphene Supermarket, USA density 1.84 g/cm^3^ and dendritic Ni powder (Alfa Aesar, mesh −325, 99.8% purity). These mixtures were subsequently cold pressed (990 MPa) and sintered under vacuum (2.0 × 10^−6^ mbar) at 900 °C for 3 h. A thorough description of the colloidal mixing process can be found elsewhere^23^.

### Sample processing

**Figure 1:**
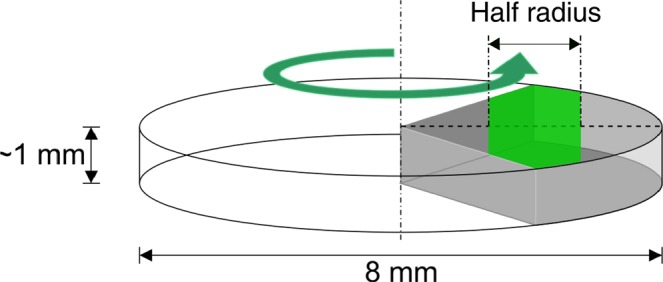
Schematic of DSC and HT-XRD test specimens taken from a HPT disc. Sample size of approximately 2 mm (along the radius ~ 1–3 mm).

Sintered samples were further processed by means of HPT. CNT/Ni composites were deformed at room temperature using different number of turns, namely 1, 4, 10 and 20 T applying 4 GPa of pressure and 0.2 rpm. The equivalent strain can be written as ${\varepsilon }_{v}\,=\,\frac{2{\rm{\pi }}{\rm{Tr}}}{{\rm{t}}\sqrt{3}}$, where T is the number of turns, t is the sample thickness and *r* is the distance from the center of the sample^24^. Different CNT fractions were used, namely 0.5, 1, and 2 wt. % (2.4, 4.7 and 9 vol. %, respectively). Pure Ni samples were also processed and analyzed as a reference state. Samples for DSC were cut in pieces containing the half radius of the samples (see Fig. 1.) using a high precision diamond wire, in order to adjust the samples to the size of the aluminum pans. The same sample preparation was implemented for the HT-XRD measurements in order to keep consistency.

### Differential scanning calorimetry

DSC was carried out using a Q2000 calorimeter (TA Instruments), using a heating rate of 15 °C/min and a Ni/Ar (50 ml/min) controlled environment, measuring the heat flow from 40 to 500 °C.

### High temperature X-ray diffraction

HT-XRD measurements were carried out using an Anton Paar HTK1200 HT-chamber at 10^-6^ mbar mounted in a PANalytical X’Pert MPD X-ray diffractometer. Diffractograms for the Ni_111_ and Ni_200_ reflections were recorded with increasing temperature as follows: room temperature, 150 °C, 200 °C and afterwards every 25 °C after 5 minutes of thermal homogenization up to 500 °C, using a heating rate of 15 °C/min.

### Transmission electron microscopy

TEM was performed by means of a JEOL JEM 2010 using 200 kV on a selected sample after DSC, namely a sample containing 2 wt. % CNT deformed by HPT using 20 T, which had shown the peak related to the decomposition of Ni_3_C.

### Electron backscattered diffraction

EBSD of the extrema processing conditions (i.e. after 1 and 20 turns) after HT-XRD was performed with a step size of 50 nm, on a dual beam system Helios NanoLab^TM^ 600 (FEI) with an attached EDAX TSL^TM^ module using 20 kV and 22 nA. Moreover, samples after annealing were also analyzed by means of EBSD using the same equipment, where the step size was varied according to the respective grain size, so that at least 6 points were to be measured within a grain. The data were processed using the EBSD data analysis software OIM 7^TM^, whereby a region of at least two adjacent points with a maximum misorientation angle of 5° was defined as a grain. Furthermore, a confidence index (CI) standardization across grains was performed, and noisy data, using a cut-off of CI = 0.09 were removed. For grain size calculations, edge grains were excluded from the analysis.

## Results and Discussion

### Differential scanning calorimetry

**Figure 2:**
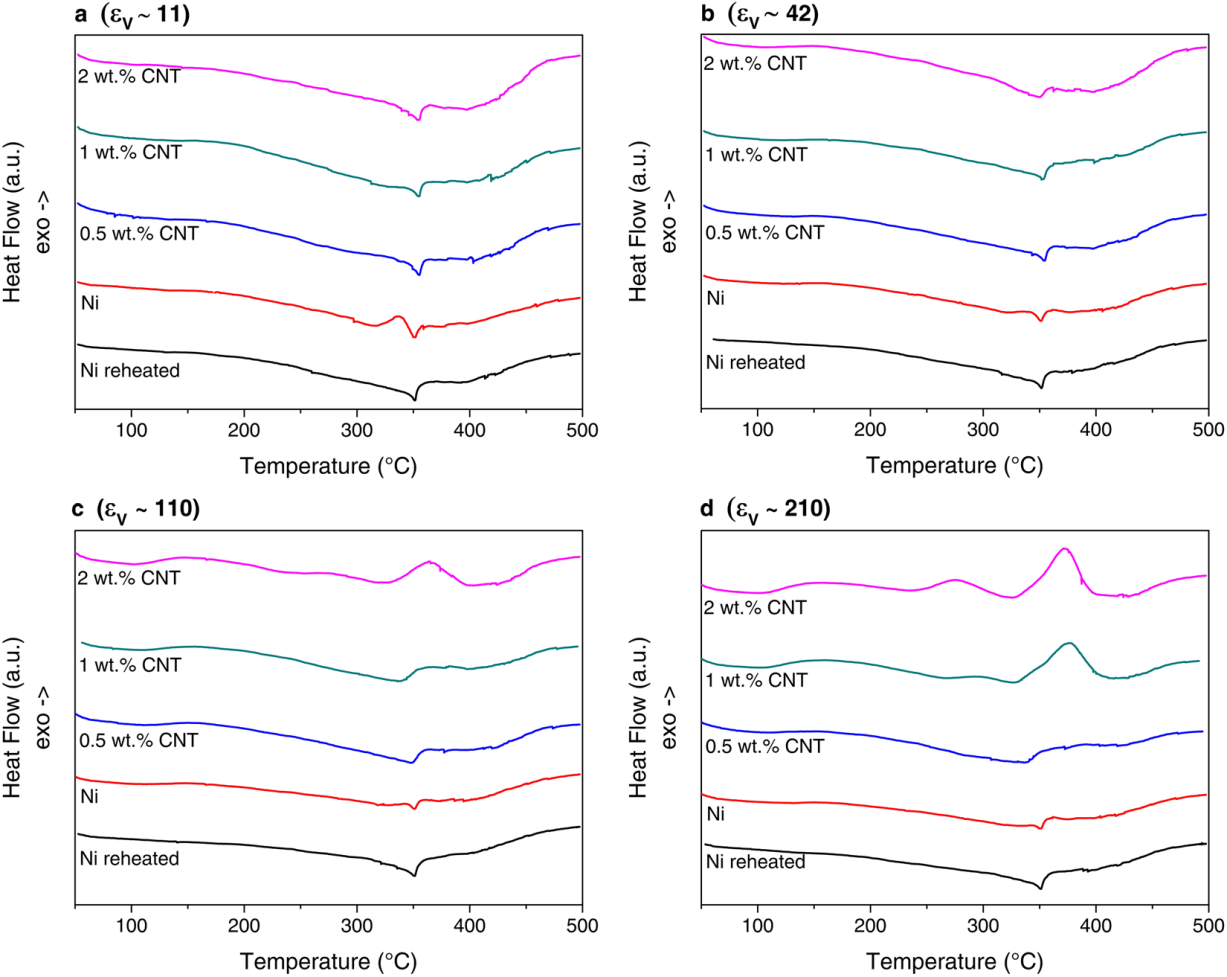
DSC curves recorded for a temperature range of 40–500 °C and a heating rate of 15 °C/min. After HPT at RT with (**a**) 1 T, (**b**) 4 T, (**c**) 10 T and (**d**) 20 T. Respective applied equivalent strains shown in each figure.

Figure 2 shows the evolution of the DSC curves for the Ni samples and the composites for the different deformation conditions. Curves of reheated Ni samples are also displayed in order to illustrate the reversible changes occurring during DSC. It can be observed that different thermal events took place during heating. The appearance of two exothermic peaks in the ranges of 100–180 °C and 200–320 °C was reported in DSC curves of Ni processed by HPT^4,5^. The first peak corresponds to the annealing of vacancies and the second to the annihilation of dislocations and activation of recrystallization^4^. Moreover, the peak related to the annihilation of dislocations overlaps with the annihilation of vacancy agglomerates and shifts to lower temperatures while the vacancy peak remains more or less constant with increasing strain^5^. Furthermore, there is an endothermic spike around 350 °C, which corresponds to the magnetic transition of Ni (i.e. the Curie temperature). The composites containing the region deformed to ε_ν_ ∼ 11 and ε_ν_ ∼ 42, behaved in a similar manner and in contrast to the corresponding Ni samples (see Fig. 2a,b), the composites showed a much lower energy release regarding the dislocation peak, which evidences their better thermal stability by the presence of CNT. With increasing strain, Ni samples also showed a lower energy release (Fig. 2b–d), which may be related to dynamic recovery processes having taking place during deformation, and/or static recovery taking place upon unloading of the samples.

**Figure 3:**
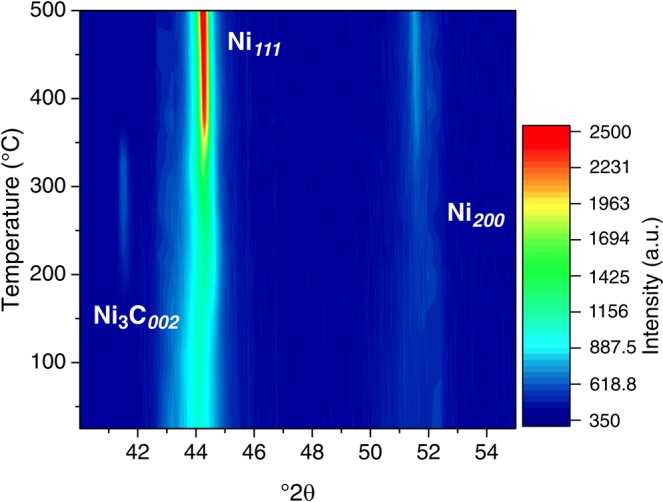
Color-coded plot of the sample 2 wt. %CNT deformed by HPT with an applied equivalent strain of ε_ν_ ∼ 210, showing the evolution of the studied 2θ range during HT-XRD.

In addition, an exothermic event appears in some composites (ε_ν_ ∼ 110: 2 wt. % CNT; ε_ν_ ∼ 210: 1 wt. % CNT and 2 wt. % CNT. see Fig. 2c,d) at around 350 °C. This exothermic peak is attributed to the decomposition of nickel carbide (Ni_3_C). Such peak has been previously observed in the course of decomposition of Ni_3_C^25,26^. Furthermore, the presence of the metastable Ni_3_C was detected between 200 °C and 350 °C during HT-XRD performed in the same samples (see Fig. 3). Since the formation of this metastable carbide is of endothermic nature, it might affect the observation of other exothermic events happening within this temperature range.

### High temperature X-ray diffraction

**Figure 4:**
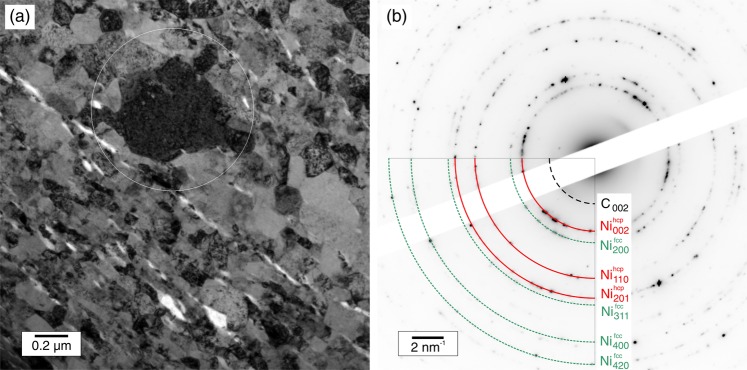
(**a**) TEM micrograph and (**b**) SAED pattern of the region marked in a, performed on the sample 2 wt. %CNT deformed by HPT with an applied equivalent strain of ε_ν_ ∼ 210, after DSC.

HT-XRD offers the possibility of studying *in-situ* the microstructural changes and phase transitions during heating. One important observation from *in-situ* XRD results is the formation and dissolution of Ni_3_C in the corresponding above mentioned composites. The formation of nickel carbide was observed in the course of deformation by ball milling of Ni-C mixtures^27^. Nevertheless, in the present study no carbide signal was detected from XRD in the initial state after HPT^22^. However, it is expected that sufficient C atoms may be available in the composites after HPT as impurities, being a consequence of irreversible damage introduced on the CNT during processing^20^. In the course of heating of the corresponding composites, a peak appears at a 2-Theta of ~41.5° between 200 to 350 °C (see Fig. 3). This XRD peak was assigned to Ni_3_C and disappears above 350 °C, which corresponds to the onset temperature of carbide decomposition (see Fig. 2c,d) when it dissolves (Ni_3_C → 3Ni + C). Ni_3_C has a metastable nature and decomposes at sufficiently high temperatures probably due to an increase of surface diffusivity and weak nature of Ni-C bond^28^. Further examination by means of TEM (see Fig. 4a,b) enabled the identification of regions containing a stabilized Ni hcp, (whose signal is marked in red in Fig. 4b), which usually acts as a witness phase of the formation and subsequent degradation of the Ni_3_C^26^.

Another feature in Fig. 3 is the shifting of the Ni_111_ peak. During heating, the XRD lines shift to the left due to lattice thermal expansion. Nevertheless, the initial Ni_111_ peak position was located at a lower angle in the as deformed state, with respect to the theoretical Ni_111_ position, most probably due to residual stresses introduced during the HPT, which are released upon heating while the peak shifts to the right. Moreover, most of the stress is released up to 200 °C and at higher temperatures the peak does not significantly shifts most likely because of stress release and thermal expansion occurring simultaneously.

**Figure 5:**
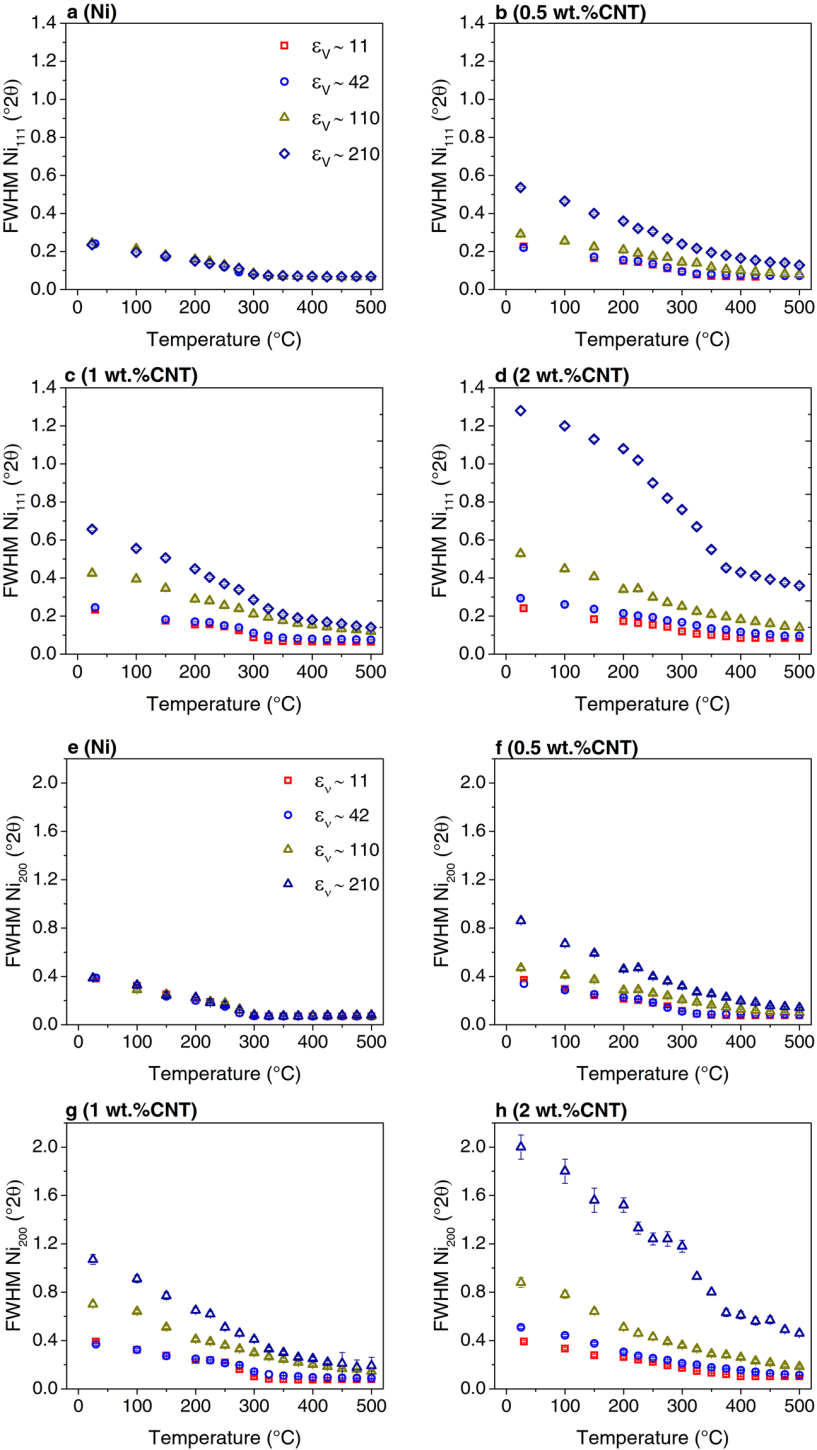
Evolution of the Ni_111_ (**a**–**d**) and Ni_200_ (**e**–**h**) reflections during heating by means of HT-XRD, recorded from 25 to 500 °C with a heating rate of 15 °C/min.

**Figure 6:**
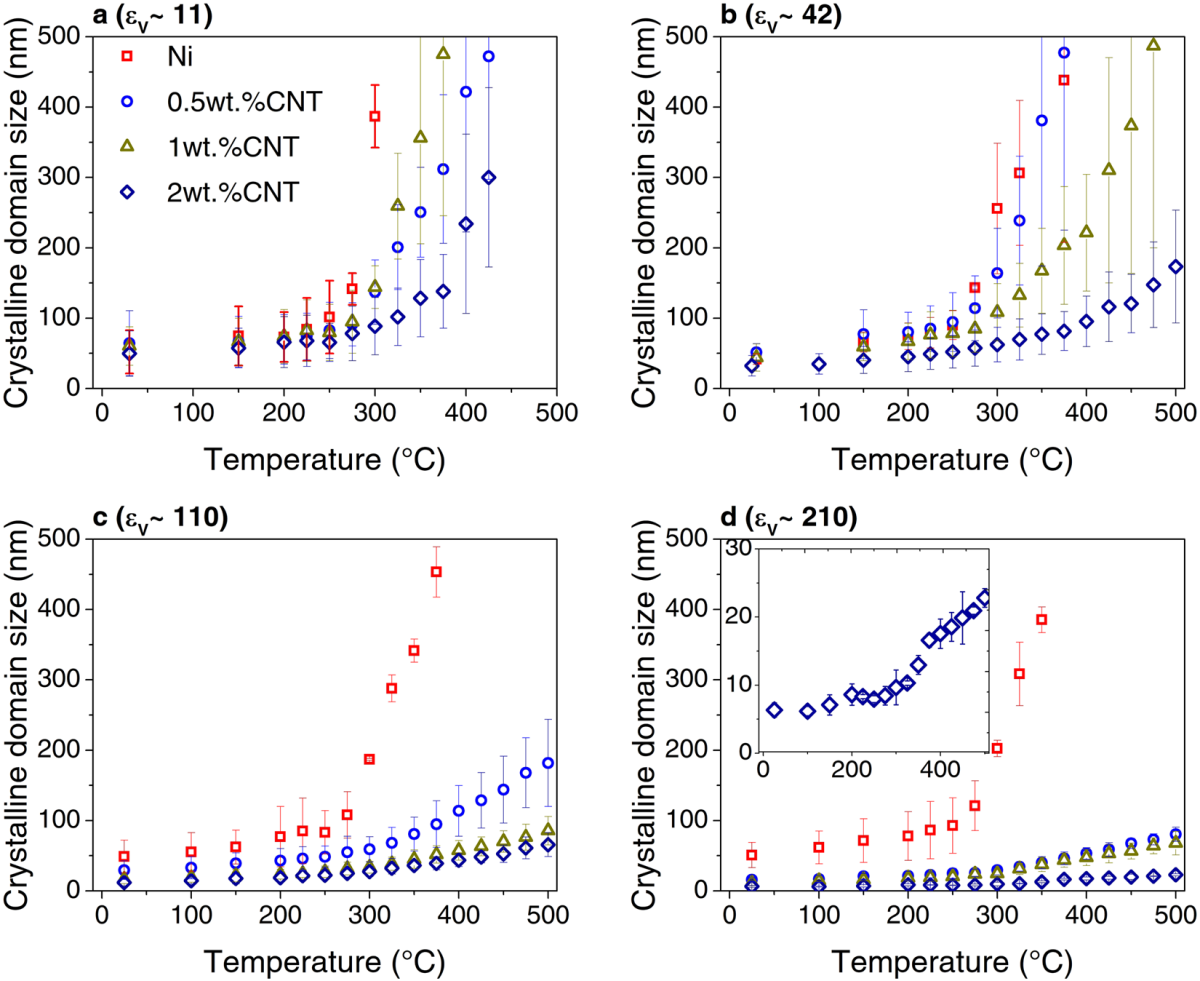
Average crystalline domain size evolution with temperature in samples deformed to (**a**) ε_ν_ ∼ 11, (**b**) ε_ν_ ∼ 42, (**c**) ε_ν_ ∼ 110 and (**d**) ε_ν_ ∼ 210.

**Figure 7:**
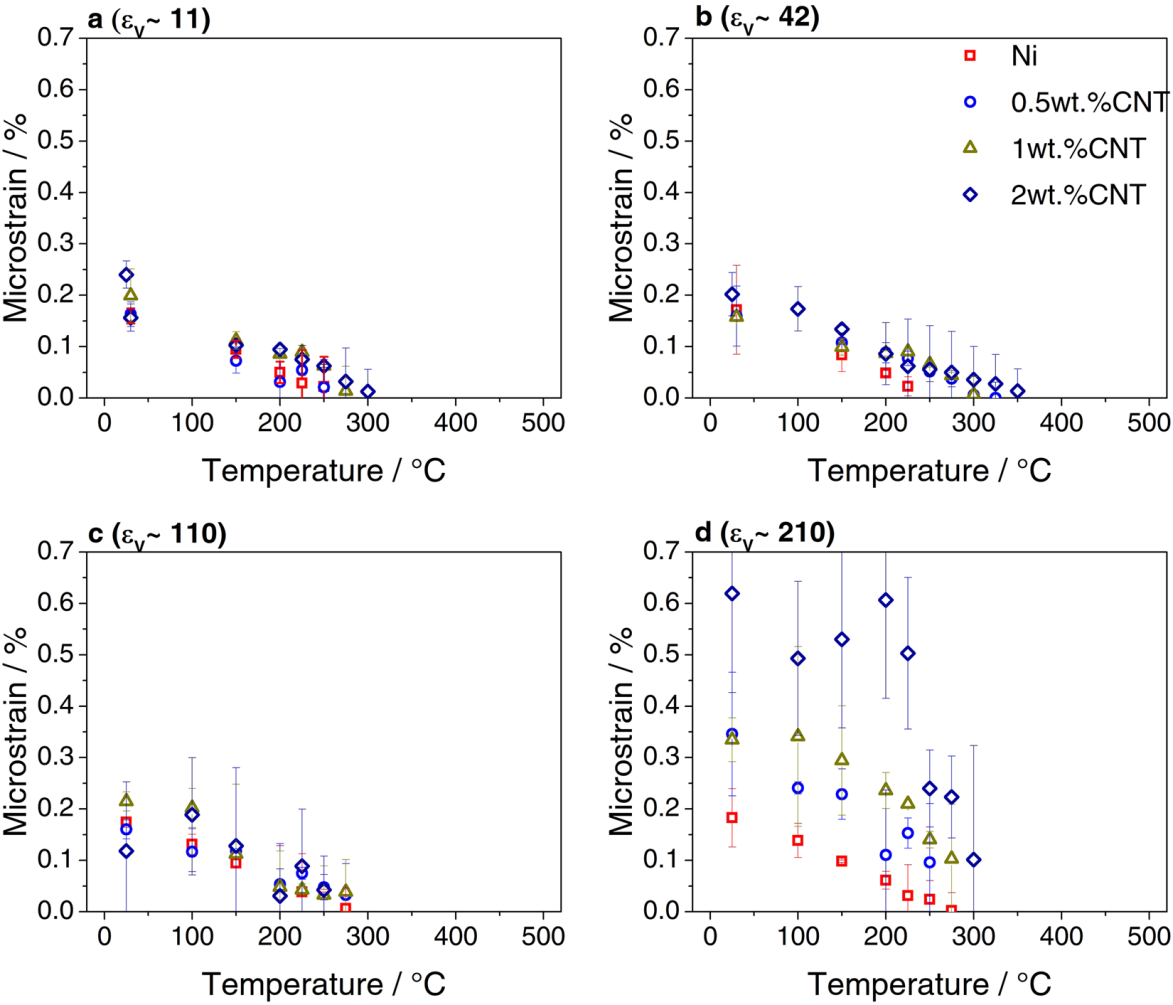
Average microstrain evolution with temperature in samples deformed to (**a**) ε_ν_ ∼ 11, (**b**) ε_ν_ ∼ 42, (**c**) ε_ν_ ∼ 110 and (**d**) ε_ν_ ∼ 210.

Furthermore, x-ray line profile analysis (XPA) can be performed in order to extract information about the evolution of the crystalline domain size and the microstrain (See supplementary information for further details). In the present study only the first two Ni reflections were measured (i.e. Ni_111_ and Ni_200_), because during isothermal holds microstructural changes occur continuously making the time for each XRD measurement very limited. Therefore, line profile analysis using the whole profile, in methods such as Whole Powder Pattern Modelling (WPPM)^29^, cannot be used in this case. According to this, the integral breadth method using single-line analysis^30^ was used in order to estimate the crystalline domain size and the microstrain changes during heating of the studied composites with respect to the Ni samples. In this regard, Fig. 5a–h shows the evolution with temperature of the measured full width at half maximum (FWHM) for the peaks Ni_111_ and Ni_200_, respectively. Ni samples behave all in the same manner and reach the minimum FWHM at about 300 °C. These results suggest that regardless of the initial accumulated imposed strain during HPT in bulk Ni samples and of the initial stored energy (see Fig. 2), there is no change in the recovery rate, at least as detected by the changes in the XRD line broadening (see Fig. 5a–e). In fact, grain growth start to happen at 250 °C in all the Ni samples (see Fig. 6). Nevertheless, the minimum microstrain, as estimated by XPA, was reached at higher temperatures in samples containing the region deformed to ε_ν_ ∼ 110 and ε_ν_ ∼ 210 (~280 °C), which also had lower energy release (see Fig. 2c,d), with respect to samples ε_ν_ ∼ 11 and ε_ν_ ∼ 42, in which the minimum microstrain occurs at ~250 °C (see Fig. 7).

Moreover, it was shown in a previous study that increasing the equivalent strain and the CNT content leads to a decrease in the crystalline domain size and an increase in the dislocation density (associated to microstrain)^22^. Accordingly, the FWHM increases with equivalent strain and CNT content (Fig. 5). In the composites deformed to equivalent strains ε_ν_ ∼ 11 and ε_ν_ ∼ 42, the evolution with temperature of the FWHM does not change significantly in samples with the same CNT content. Nevertheless, the grain growth rate (analogous to crystallite size) decreases with increasing CNT content (see Fig. 6), and it takes place when the recovery (as described by the decrease in microstrain with increasing temperature) has finished (see Fig. 7). In other words, the recrystallization and grain coarsening is retarded while the recovery process takes place, i.e. during annealing and rearrangement of dislocations. Furthermore, the carbide formation has an effect on the evolution of both the crystalline domain size and the microstrain. In fact, it can be observed in the insert of Fig. 6d, which is a magnified image on the evolution of crystallite size for the sample 2 wt. % CNT, that between 200–350 °C the growth rate is zero and afterwards increases. Additionally, between 100–200 °C there is a slight increase in the microstrain and after 200 °C the microstrain decreases quickly (also note the respective FWHM changes in Figs. 5,d and 5h). This can be related to an increase in the amount of dislocations, which are being generated during the formation of the carbide probably due to CTE mismatch between the carbide and the nickel matrix, and while the microstrain decreases, the growth rate is retarded.

**Figure 8:**
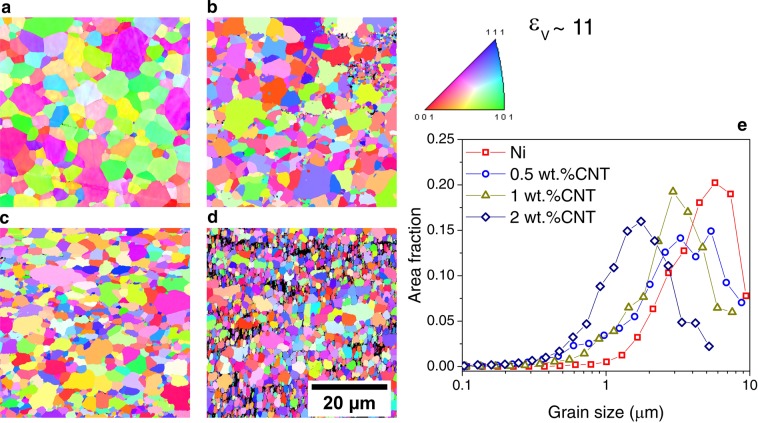
Inverse pole figures of samples deformed by HPT with ε_ν_ ∼ 11 after ramp annealing (by HT-XRD in) (**a**) Ni, (**b**) 0.5 wt. %CNT, (**c**) 1 wt. %CNT and (**d**) 2 wt. %CNT; **e**) Grain size distributions of the same samples. Scale shown in d is common to all the images.

**Figure 9:**
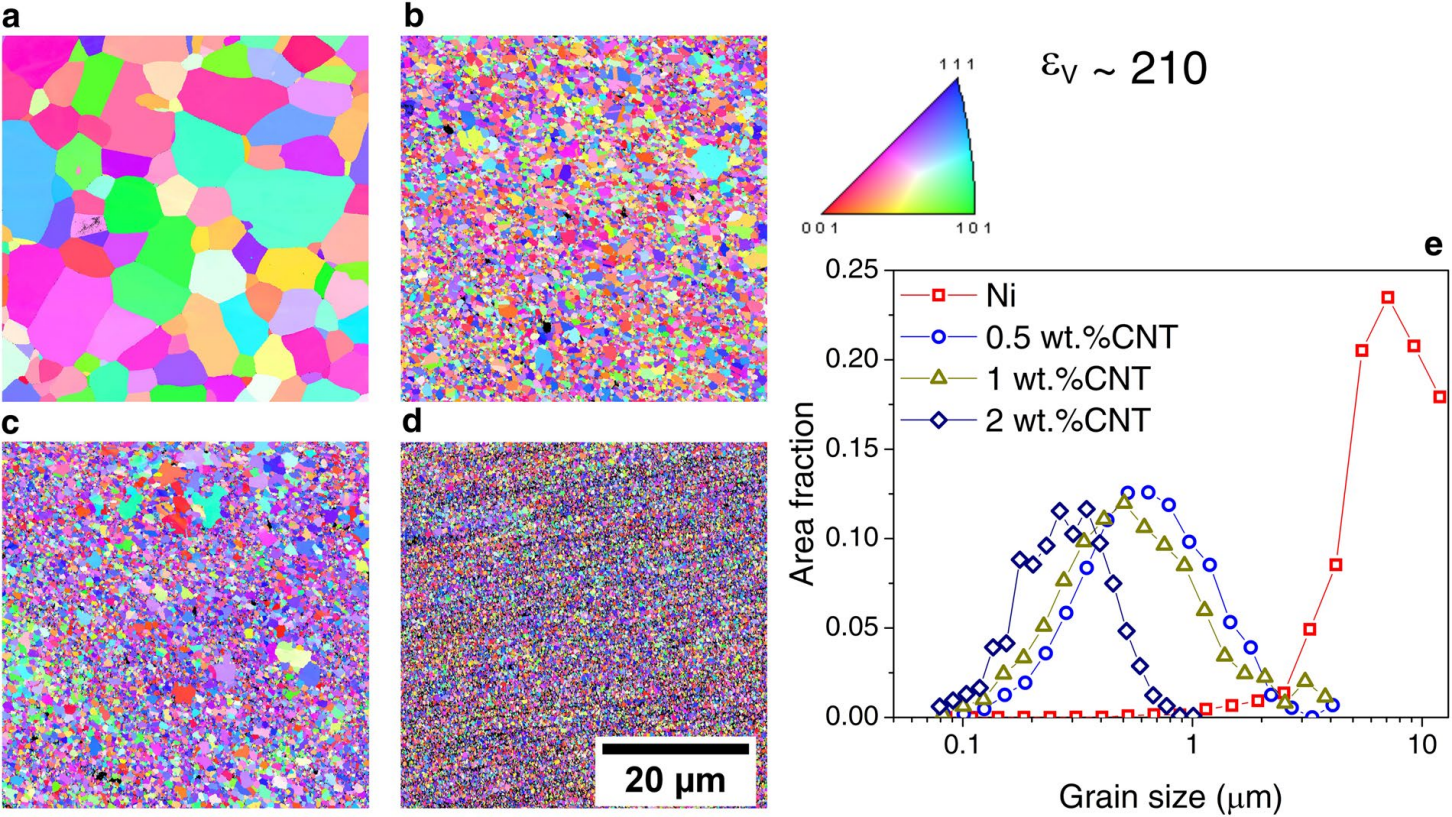
Inverse pole figures of samples deformed by HPT with ε_ν_ ∼ 210 after ramp annealing (by HT-XRD) in (**a**) Ni, (**b**) 0.5 wt. %CNT, (**c**) 1 wt. %CNT and (**d**) 2 wt. %CNT; (**e**) Grain size distributions of the same samples. Scale shown in d is common to all the images.

Samples deformed to ε_ν_ ∼ 11 (see Fig. 8a–d) and to ε_ν_ ∼ 210 (see Fig. 9a–d) were analyzed by means of EBSD in order to assess the final grain size and possible characteristic features after annealing by HT-XRD. From the grain size plots, displayed in Figs. 8,e and 9e, it can be observed that the composites resulted in a finer microstructure than the respective Ni samples. This effect is more pronounced in the composites with higher initial equivalent strain, which presented an annealed UFG microstructure. In order to further study the microstructural stability of the composites, isothermal annealings were performed.

### Isothermal annealing

**Figure 10:**
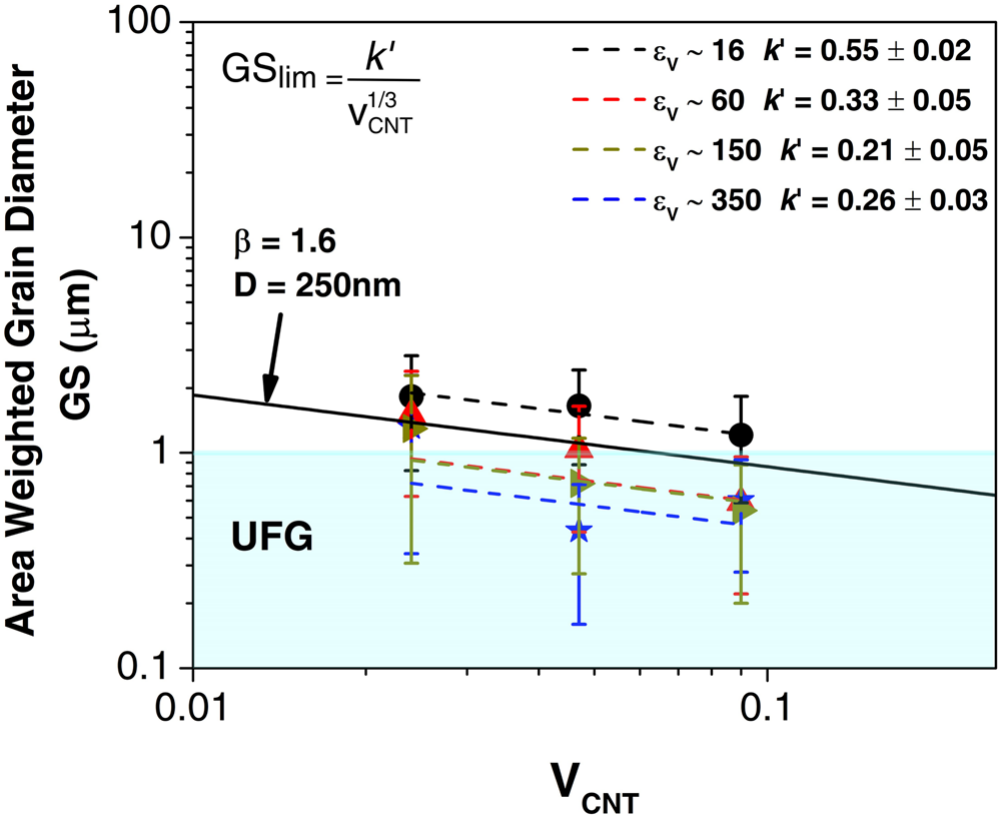
Symbols correspond to the average grain sizes obtained from EBSD after 12 h annealing at 500 °C. The dashed lines correspond to the respective Zener model. The UFG region is shown. The values for k’ for the corresponding equivalent strains are presented in the graphic. A reference line is plotted using an average CNT agglomerate size D = 250 nm and a geometric constant of β = 1.6 as in ref. ^32^.

*Zener pinning model*. Second-phase particles decrease the maximum grain size obtained after annealing by kinetic stabilization of the grain boundaries^31^ (a phenomenon known as “Zener drag”). The limiting grain size can be written as $G{S}_{lim}=\frac{k\text{'}}{{f}^{m}}$, where k’ and m are constants and f is the volume fraction of pinning particles (i.e. CNT agglomerates). Furthermore, k’ comprises a constant β and the particle size D. For large volume fractions (f ~ 0.02) and for particles located mainly at the grain boundaries m = 1/3^32,33^. The composites in the present study were fabricated by powder metallurgy (solid state processing) and consequently, the CNT are expected to be present only along the grain boundaries. The Ni samples and the composites were annealed during 12 h at 500 °C, which corresponds to a homologous temperature of 0.45T_m_, and the final grain size was measured by means of EBSD at 3 mm from the samples’ centers along the radial direction. Figure 10 shows the composites’ final grain sizes (symbols) together with the respective Zener models $G{S}_{lim}=\frac{k\text{'}}{{V}_{CNT}^{1/3}}$, where V_CNT_ is the CNT volume fraction used for the fabrication of the composites. Results for Ni samples can be found in Table S1 from the supplementary material. It should be mentioned that the CNT agglomerates are expected to be more uniformly dispersed with higher equivalent strains and their final agglomerate sizes lie within the same range^21^. Within experimental uncertainty, the composites showed a similar behavior with final grain sizes lying rather within the UFG range. Moreover, it can be observed that the curves adjust well to m = 1/3. Nevertheless, there are certain differences in grain size among samples deformed with different equivalent strains, which can be related to several phenomena taking place during annealing. For instance, it is well known that finer microstructures will recrystallize faster but are expected to result in smaller grain sizes. Also, the recrystallization process in SPD materials can be inhomogeneous due to heterogeneity in the as deformed structure being transferred into the annealed samples, for example, because HAGB have higher mobility than LAGB and regions with higher amounts of HAGB recrystallize first. Furthermore, grains located in regions with higher stored energy grow to a larger size. Additionally, the final grain size is also affected by the way how the agglomerates are dispersed and a non-homogeneous distribution could lead to abnormal grain growth with some grains being pinned and not others.

## Conclusions

Ni/CNT composites processed by HPT exhibited an improved thermal stability upon annealing for 12 h at a homologous temperature of 0.45T_m_ with final grain sizes dependent on the CNT volume fraction following an f ^−1/3^ relationship consistent with theoretical Zener drag models. Furthermore, the microstructural changes during heating and therefore, the final microstructure, were affected by the initial microstructural state of the samples and the CNT after SPD. Thereby, the formation of nickel carbide was detected by DSC and HT-XRD and the evolution of the microstructure was analyzed by means of X-ray line profile analysis. Even though irreversible damage is introduced to the CNT, these are able to pin the grain boundaries during grain growth, and although recovery of defects and grain growth took place during heating, the composites presented a final ultrafine microstructure. It can be thus concluded that the use of CNT in NC and UFG materials processed by SPD successfully stabilizes the microstructure after heating. The present work provides relevant understanding on the assessment of the thermal stability in CNT-MMC and can be useful for further research in the area of SPD of engineering materials.

## Supplementary information

Supplementary Information.

## Data Availability

The datasets used in the present study may be made available on reasonable request.
