# Supplementary material for: Microstructural evolution during heating of CNT/Metal Matrix Composites processed by Severe Plastic Deformation
Source: Sci Rep. 2020 Jan 21;10:857. doi: 10.1038/s41598-020-57946-3 (PMC6972703; doi:10.1038/s41598-020-57946-3)
Supplement: Supplementary file 1 — Supplementary Information. [file 41598_2020_57946_MOESM1_ESM.pdf]

Microstructural evolution during heating of CNT/Metal Matrix Composites processed by Severe Plastic Deformation

Katherine Aristizabal[1, *], Andreas Katzensteiner[2], Andrea Bachmaier[2], Frank Mücklich[1] and Sebastian Suárez[1,*].

[1]Chair of Functional Materials, Department of Materials Science, Saarland University. D-66123, Saarbrücken, Germany.

[2]Erich Schmid Institute of Materials Science, Austrian Academy of Sciences, Jahnstrasse 12, A-8700 Leoben, Austria.

Supplementary information

X-ray single line profile analysis.

Different methods for the determination of the structural parameters by means of X-ray diffraction rely on the analysis of as many reflections available for the determination of the integral breadth (e.g. Williamson-Hall and the respective modified versions), or rely on at least two harmonic reflections (e.g. Warren-Averbach and the respective modified versions). Nevertheless, when only one Bragg peak, for example, is available from experimental results, single line analysis provides an appropriate approach, which is based on experimental observations that crystallite size causes Cauchy-type broadening (i.e. Lorentzian) while strain broadens the peak following a Gauss profile. Thereby, the Cauchy and Gauss fractions of a Bragg reflection represent the size and the strain contributions, respectively[1,2]. By using a Pseudo-Voigt type line profile convolution of the available Bragg peaks, the Cauchy and the Gauss parts of the instrument and the sample are obtained

and after extracting the instrumental contribution, the integral breadths (B) of the sample's profile broadenings are used to calculate the average crystallite size $<D>_V$ and the weighted average strain $\bar{\varepsilon}$ in [%] (mean lattice distortion), according to:

$$< D >_V = K\lambda / B_{Size} cos\theta$$

$$\bar{\varepsilon} = B_{Strain} / 4tan\theta$$

Where λ is the X-ray wavelength; K is a shape factor, which changes depending of the assumed shape. Thus, for spheres it is 1.07 and for cubes it ranges between 1 and 1.16. In the present study, a shape factor of 1 was used. Furthermore, the crystalline domain size and the microstrain reported correspond to peak-area weighted averages of the Ni_{111} and Ni_{200} Bragg peaks.

Table S 1

Grain sizes for Ni samples measured by EBSD after 12h annealing at 500°C.

Equivalent strain	Mean grain size in µm
16	8.44 ± 4.68
60	6.69 ± 4.14
150	5.78 ± 3.20
350	5.01 ± 2.48

References

1. De Keijser, T., Langford, J. I., Mittemeijer, E. J. & Vogels, A. B. P. Use of the Voigt function in a single-line method for the analysis of X-ray diffraction line broadening. *J. Appl. Crystallogr.* **15**, 308–314 (2002).

2. De Keijser, T., Mittemeijer, E. J. & Rozendaal, H. C. F. The determination of crystallite-size and lattice-strain parameters in conjunction with the profile-refinement method for the determination of crystal structures. *J. Appl. Crystallogr.* **16**, 309–316 (1983).
